# Associations between infant growth and pubertal onset timing in a multiethnic prospective cohort of girls

**DOI:** 10.1186/s12887-022-03242-0

**Published:** 2022-03-31

**Authors:** Sara Aghaee, Charles P. Quesenberry, Julianna Deardorff, Lawrence H. Kushi, Louise C. Greenspan, Assiamira Ferrara, Ai Kubo

**Affiliations:** 1 Kaiser Permanente Northern California Division of Research, 2000 Broadway, Oakland, CA 94612 USA; 2 Division of Maternal and Child Health, University of California, School of Public Health, 2121 Berkeley Way #5302, Berkeley, CA 94720 USA; 3 Kaiser Permanente San Francisco Medical Center, 2425 Geary Boulevard, San Francisco, CA 94115 USA

**Keywords:** Adolescent health, Infant development, Childhood overweight, Puberty

## Abstract

**Background:**

Early puberty increases risk of adverse health conditions throughout the life course. US girls are experiencing earlier puberty without clear reasons. Studies suggest early life factors, such as infant growth, may influence pubertal timing. We assessed the associations between infant growth and onset of breast development (thelarche), pubic hair development (pubarche), and menarche in girls.

**Methods:**

A prospective cohort of girls born at a Kaiser Permanente Northern California medical facility in 2005–11 was used. Weight-for-age z-scores were calculated at birth and 24 months. Difference in z-scores greater than 0.67 represent rapid “catch-up” growth, less than -0.67 represent delayed “catch-down” growth, and between -0.67 and 0.67 represent “normal” growth. Pubertal onset was measured using clinician-assessed sexual maturity ratings (SMRs) and defined as the age at transition from SMR 1 to SMR 2 + for both thelarche and pubarche. SMR data was collected through June 2020. Menarche was analyzed as a secondary outcome. Weibull and modified Poisson regression models were used. Models were adjusted for potential confounders.

**Results:**

There were 15,196 girls included in the study. Approximately 30.2% experienced catch-up growth, 25.8% experienced catch-down growth, and 44% had normal growth. Girls with catch-up growth had increased risk of earlier thelarche (hazard ratio = 1.26, 95% confidence interval (CI): 1.18, 1.35), pubarche (1.38, 95% CI: 1.28, 1.48), and menarche (< 12y, relative risk = 1.52, 95% CI: 1.36, 1.69) compared to those with normal growth, after adjusting for covariates. These associations were partially mediated by childhood body mass index. Catch-down growth was associated with later pubertal onset.

**Conclusions:**

Girls who experience infant catch-up growth have higher risk of earlier pubertal development compared to girls with normal growth and the associations are partially explained by childhood obesity. This information may help clinicians to monitor girls who are at high risk of developing earlier.

## Background

Girls in the U.S. are experiencing puberty earlier, compared with just a few decades ago [1]. This trend is an important public health concern because early puberty in girls is associated with higher risks of adverse mental and physical health conditions throughout the life course [2–5]. Childhood obesity is a known predictor of pubertal timing; however, it alone does not explain the trend toward earlier puberty as children with normal body mass index (BMI) are also experiencing similar trends [6]. Because sexual developmental events are part of a continuum that begins during intrauterine life, perinatal factors likely influence the programming of pubertal maturation [7].

Previous studies have reported that infant catch-up growth is associated with earlier puberty [8–13]. However, these studies have several methodological limitations. First, many of these studies focused on self-reported age at menarche. Girls’ pubertal transitions take place over several years, often starting with thelarche, typically followed by pubarche, acne, a growth spurt, and then menarche. Despite occurring late in the pubertal process, menarche is often used as the only pubertal marker. Recent studies have reported that timing of thelarche and pubarche may be more important risk factors for adverse outcomes such as depression, substance abuse, and delinquency in adolescents [14–16] than age at menarche, thus examining pubertal onset as an outcome is also important. Second, few previous studies have used clinician-assessed sexual maturity ratings (SMRs), also known as Tanner stages, an established gold standard 5-point staging system for breast and pubic hair development [17]. Among previous studies that used SMRs, only one conducted in China used clinician-assessed SMRs [18], while others were self-reported [10, 13]. Lastly, many studies did not include important covariates of infant weight gain and pubertal development, such as maternal gestational weight gain (GWG), prior livebirths, or childhood obesity [19–21]. Since infant catch-up growth is strongly associated with GWG [19, 20] and childhood obesity [22, 23], it is important to include these variables in the analysis. We addressed these limitations by conducting a prospective study using a large, racially/ethnically diverse cohort of girls from Northern California and comprehensive clinical data.

## Methods

### Cohort selection

A birth cohort of mother-daughter pairs were identified from the Kaiser Permanente Northern California (KPNC) electronic health record (EHR) system and were followed until June 30, 2020. KPNC is an integrated health care delivery system serving over 4.4 million members in Northern California. KPNC members are representative of the general population of Northern California with regard to ethnicity and education [24, 25]. Eligibility for the girls included: singleton and full-term (> 36 weeks gestation) birth at a KPNC medical facility between 2005 and 2011, continuous KPNC membership during the follow-up period, with coverage gaps of ≤ 90 days, availability of childhood BMI measurement (5–6 years old), at least one SMR assessment (details described below) between ages 5 and 18 years, and weight measurements at birth and within two weeks of their second birthday. Exclusion criteria include: girls with medical conditions affecting growth and development, such as adrenal tumors and congenital adrenal hyperplasia (*n* = 478) and mothers with extreme BMI (< 15 kg/m^2^ or > 60 kg/m^2^) (*n* = 12).The final cohort consisted of 15,196 mother-daughter pairs. All the data were obtained from the KPNC EHR system and administrative databases.

### Exposure

Infant growth was measured as weight trajectory patterns and calculated using change in weight-for-age z-scores between birth and 24 months. Z-scores were determined using age- and sex-specific Centers for Disease Control and Prevention year 2000 standard population distributions [26]. A change in z-score was categorized as “catch-up” growth when > 0.67; “catch-down” growth when < -0.67; and “normal” growth for change in z-scores ranging between -0.67 and 0.67. A z-score of 0.67 has been used previously to indicate clinically-significant catch-up or catch-down growth [27].

### Outcomes

Starting in 2010, KPNC pediatricians and family physicians began documenting in the EHR 5-point SMR[17] (1 = no development; 5 = full maturation) as part of routine pediatric checkups starting at age 6 years. At KPNC, breast SMRs are determined using palpation and visual inspection, while pubic hair SMRs are assessed using visual inspection. We have confirmed the validity of using the KPNC EHR system SMR data in a previous study [28]. In the present study, our primary outcomes of interest were age at transition from SMR 1 (prepubertal) to SMR 2 + for breast (thelarche) and pubic hair (pubarche). Given the study design, we are unable to observe the exact ages at these transitions. However, using the information obtained from observed SMRs at routine checkups, standard statistical methods can be applied to estimate age at onset distributions and measures of association between covariates and age of thelarche and pubarche.

Girls who indicated having gotten their menses before 12 years, the current average age of menarche in the United States, were categorized as having ‘earlier’ menarche [29]. Menarche data was collected using responses from KPNC health check-up questionnaires. Questions about menses are asked at Well-Child 10–12 Years (“Has your daughter started menstruating?”) and Well-Teen (“Have you started your period?”) check-ups. Girls were considered to have earlier menarche if they responded “Yes” before age 12 years. Girls who responded “No” before or after 12 years were considered to have normal/later menarche. Girls who responded “Yes” after age 12 years and had no data before 12 years were excluded from analyses to prevent misclassification.

### Covariates

BMI percentiles [26] were calculated using girls’ weight and height obtained from clinic visits between the ages 5 and 6 years old.

In our analyses, we adjust for clinically relevant covariates that have been associated with infant anthropometric measurements and later pubertal development. These include GWG [30, 31], maternal age at delivery (years) [32, 33], socioeconomic status (maternal education [high school or less, some college, college graduate, postgraduate]) [34, 35], prior livebirths (0, 1, 2 +) [36, 37], breastfeeding duration (never, < 6 months, ≥ 6 months) [38, 39], girl’s race/ethnicity (White, Black, Hispanic, Asian/Pacific Islander, and other/unknown) [1, 37], birthweight (grams) [40, 41], and gestational age (weeks) [32, 37]. Maternal GWG was calculated as delivery weight (kg) minus periconceptional weight (kg). Periconceptional weight is the weight measured closest to conception and was selected from recorded weights up to a year prior to conception. Delivery weight was measured closest to the delivery date within 45 days prior to delivery. GWG was categorized in accordance with Institute of Medicine guidelines as “below”, “excessive” or “met” [42].

### Statistical analysis

**Fig. 1:**
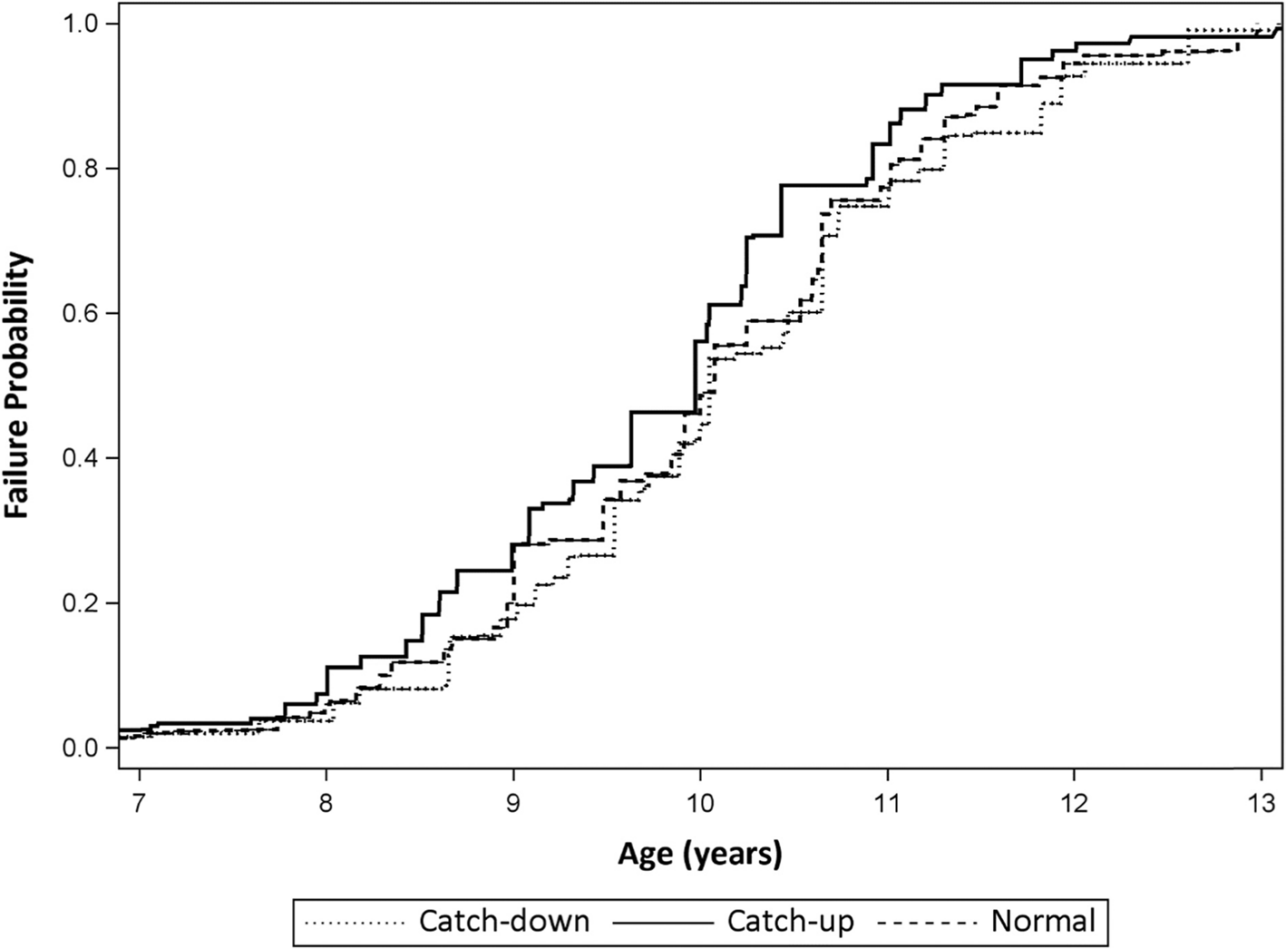
Probability of Experiencing Thelarche by Age and Infant Growth Patterns: KPNC Puberty Study (2010–2020)

**Fig. 2:**
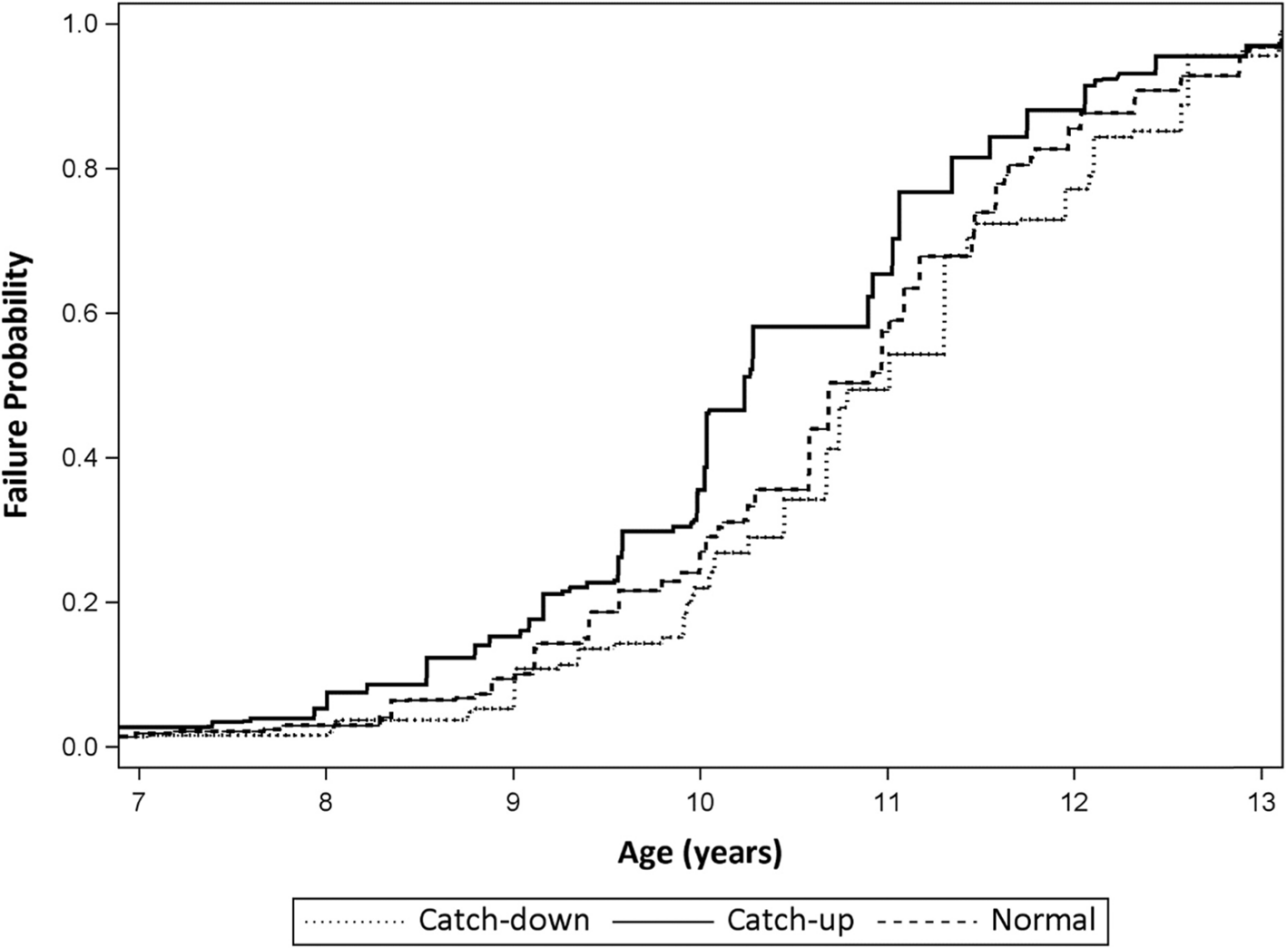
Probability of Experiencing Pubarche by Age and Infant Growth Patterns: KPNC Puberty Study (2010–2020)

Nonparametric estimates of the cumulative distributions of age at pubertal onset were calculated and stratified by infant growth category (Figs. 1 and 2) [43]. Analyses of infant growth in relation to age of thelarche and pubarche, with covariate adjustment, used parametric survival (Weibull) regression models, providing maximum likelihood estimates of hazard ratios and time ratios (TR) with 95% confidence intervals [44].TR estimates represent the ratio of the median time to event for a given level of the exposure variable in relation to its reference level. Estimation of age at onset distributions and Weibull regression parameters accommodated left, right and interval censoring. Girls were considered left-censored if they had already transitioned to SMR 2 + at the time of the first SMR exam. They were right-censored at the time of their last exam if they had not transitioned to SMR 2 + or had only 1 assessment at SMR 1. Girls who had an exam with an assessment of SMR 1 and a later assessment of SMR 2 + were considered interval-censored, as the exact age at transition between the SMR 1 and 2 + assessments is unknown. Such censoring is a commonly encountered problem which is inherent to study designs where the presence of an event can only be assessed periodically (e.g. at clinic visits spaced over time), and it is a feature of most studies of pubertal transitions. Statistical estimation and inference techniques for censored time to event data are standard and well-developed, and we have applied such procedures in our analyses. Given that censoring only impacts the approach to estimation of the cumulative age distribution and regression model parameters (e.g. hazard ratio), interpretation of analysis results is the same whether there is or is not censoring during cohort follow-up.

Modified Poisson regression models were used to model menarche comparing earlier menarche (age < 12 years) to normal/later menarche (referent), for point and interval rate ratio estimation (i.e. ratio of proportions with earlier menarche). All the models were adjusted for the same covariates as above.

As a secondary analysis, we considered whether changes in weight at particular age periods were more likely to be associated with age at pubertal onset. We examined weight changes from birth to 2 months, 2 to 9 months, and 9 to 19 months by examining associations between continuous change in weight for age z-scores at each timepoint with each outcome (thelarche, pubarche, menarche) in separate models using girls with measurements in all three time intervals. We also examined race/ethnicity and GWG as potential effect modifiers by using a cross-product term of each variable with infant growth patterns. Additional secondary analyses assessed the mediating effects of childhood BMI (percentiles), with point and interval estimation of the percent of the infant growth effect mediated by BMI, expressed as the ratio of the natural indirect infant growth effect divided by the total effect. The natural indirect effect through a mediator is quantified by using regression models to estimate how infant growth affects childhood BMI, and how this change would in turn affect age at pubertal transition. Estimates are provided for the effects of catch-up growth vs normal and for catch-down growth vs normal assuming the Weibull regression model for infant growth and childhood BMI in relation to age at pubarche and thelarche (dependent variables) with censoring, and a linear regression model relating infant growth to childhood BMI (dependent variable) [45, 46]. All analyses were conducted using SAS version 9.4 (SAS Institute, Cary, NC).

#### Model Fit

Point and interval estimates of the gamma shape parameter indicated that the Weibull distribution assumption (gamma shape parameter = 1) is quite reasonable for analyses of pubarche, with an estimated gamma shape parameter of 1.07 (95% CI: 0.93, 1.21). The Weibull model fit in analyses of thelarche is not as good, with estimated shape parameter of 0.76 (95% CI: 0.66, 0.85). We note, however, that survival curves do not cross-over, and that our TR and HR estimates are interpreted as infant growth effects averaged over the ages of pubertal onset, with any slight departures from proportionality not impacting this interpretation. We also note that regression parameters from the more general gamma distribution are very difficult to interpret, and that fitting time-stratified Weibull regression models to obtain HR estimates for specific age-intervals in order to capture heterogeneity across age (e.g. < 8, 8–9, 9–10….) is not an option given the heavy left and interval censoring (pubertal status is unknown at the beginning of each fixed age interval). The approach taken here is reasonable, with proper interpretation as age-averaged effects.

#### Missing data

Approximately 25.1% of girls had missing information on at least one covariate: maternal education (*n* = 202, 1.3%), prior livebirths (*n* = 14, 0.1%), GWG (*n* = 2,634, 17.3%), and breastfeeding duration (*n* = 2,925, 19.2%). Multiple imputation was used for handling missingness, using the chained equation technique to generate 50 imputed datasets [47].The discriminant function method was used to impute missing values for the four categorical variables, with imputation based on the following covariates: infant growth patterns, birth year, maternal age at delivery, maternal education, prior livebirths, birthweight, gestational age, race/ethnicity, GWG, breastfeeding duration, and childhood overweight/obesity status (binary). Regression analyses, outlined above, were performed on each of the imputed datasets, with results combined using Rubin’s rules [48], providing valid point and interval estimates appropriately accounting for the uncertainty in imputing the missing data.

### Ethics approval

This project was approved by the KPNC Institutional Review Board.

## Results

### Participant characteristics

Of the 15,196 girls in the study, 30.2% experienced infant catch-up growth, 25.8% catch-down growth, and 44.0% normal growth (Table 1). Girls with catch-up growth were more likely to be obese/overweight, be non-White, and have a lower gestational age than their counterparts. Girls with catch-down growth had the highest average birthweight. Among girls with information on breast development, 14.5% were left-censored (already had transitioned to stage 2 +) and 57.8% were right censored (did not transition to stage 2 + during the study period), and among girls with pubic hair data, 10.6% and 67.1% were left- and right-censored, respectively.

**Table 1:** Distribution of Characteristics by Infant Growth Patterns: KPNC Puberty Study (2010–2020), *N* = 15,196

**Infant Growth Patterns**	**Catch-up growth** **(n=4,589)**	**Catch-down growth** **(n=3,915)**	**Normal growth** **(n=6,692)**	***P* value**
	**N (%)**	**N (%)**	**N (%)**	
Maternal Characteristics				
Age at delivery (years)^a^	30.3 (5.5)	31.0 (5.0)	30.6 (5.2)	<0.001
Gestational weight gain				
Exceeds	2,040 (44.5)	1,889 (48.3)	3,031 (45.3)	0.001
Met	1,158 (25.2)	932 (23.8)	1,730 (25.9)	
Below	596 (13.0)	417 (10.7)	769 (11.5)	
Missing	795 (17.3)	677 (17.3)	1,162 (17.4)	
Prior livebirths				
0	2,417 (52.7)	1,617 (41.3)	3,201 (47.8)	<0.001
1	1,399 (30.5)	1,531 (39.1)	2,336 (34.9)	
2+	770 (16.8)	762 (19.5)	1,149 (17.2)	
Missing	3 (0.1)	5 (0.1)	6 (0.1)	
Education				
High school or less	1,192 (26.0)	840 (21.5)	1,522 (22.7)	<0.001
Some college	1,393 (30.4)	1,124 (28.7)	1,888 (28.2)	
College graduate	1,242 (27.1)	1,232 (31.5)	1,998 (29.9)	
Postgraduate	706 (15.4)	667 (17.0)	1,190 (17.8)	
Missing	56 (1.2)	52 (1.3)	94 (1.4)	
Breastfeeding duration				
Not breastfed	403 (8.8)	220 (5.6)	414 (6.2)	<0.001
< 6 months	1,472 (32.1)	935 (23.9)	1,887 (28.2)	
≥ 6 months	1,818 (39.6)	2,016 (51.5)	3,106 (46.4)	
Missing	896 (19.5)	744 (19.0)	1,285 (19.2)	
Girl’s Characteristics				
Gestational age (weeks)^a^	38.9 (1.1)	39.5 (1.0)	39.2 (1.1)	<0.001
Birthweight (grams)^a^	3164.4 (370.4)	3645.1 (466.3)	3381.0 (394.9)	<0.001
Childhood BMI				
≥85^th^ percentile	1,963 (42.8)	522 (13.3)	1,412 (21.1)	<0.001
Race/ethnicity				
White	1,477 (32.2)	1,545 (39.5)	2,551 (38.1)	<0.001
Black	384 (8.4)	172 (4.4)	361 (5.4)	
Hispanic	1,362 (29.7)	875 (22.3)	1,613 (24.1)	
Asian/Pacific Islander	984 (21.4)	973 (24.9)	1,596 (23.8)	
Other/unknown	382 (8.3)	350 (8.9)	571 (8.5)	

*BMI* body mass index, *KPNC* Kaiser Permanente Northern California

^a^Values are expressed as mean (standard deviation)

### Primary analyses

#### Infant growth patterns and thelarche

After adjusting for confounders, girls with infant catch-up growth were more likely to experience earlier thelarche (HR: 1.26, 95% CI: 1.18, 1.35; TR: 0.97, 95% CI: 0.97, 0.98) compared with girls with normal growth (referent). This time ratio corresponds to approximately 3 months earlier breast development. On the other hand, girls with catch-down growth were more likely to experience later thelarche (HR: 0.84, 95% CI: 0.78, 0.90; TR: 1.02, 95% CI: 1.01, 1.03), or approximately 2 months *later* breast development onset, compared to the referent (Table 2).

**Table 2:** Association Between Infant Growth Patterns and Timing of Thelarche: KPNC Puberty Study (2010–2020)

		**Unadjusted**		**Adjusted** ^**a**^	
**Infant Growth Patterns**	**N**	**TR (95% CI)**	**HR (95% CI)**	**TR (95% CI)**	**HR (95% CI)**
Catch-up	4,514	0.98 (0.97, 0.98)	1.24 (1.16, 1.32)	0.97 (0.97, 0.98)	1.26 (1.18, 1.35)
Catch-down	3,854	1.01 (1.01, 1.02)	0.89 (0.83, 0.96)	1.02 (1.01, 1.03)	0.84 (0.78, 0.90)
Normal	6,586	1.00 (Reference)	1.00 (Reference)	1.00 (Reference)	1.00 (Reference)

*CI* confidence interval, *HR* hazard ratio, *KPNC* Kaiser Permanente Northern California, *TR* time ratio

^a^Adjusted for maternal age, education, prior livebirths, GWG, and girl’s birthweight, gestational age, breastfeeding duration, and race

#### Infant growth patterns and pubarche

Similar to the thelarche models, girls with catch-up growth were more likely to experience earlier pubarche (HR: 1.38, 95% CI: 1.28, 1.48; TR: 0.97, 95% CI: 0.96, 0.97), and girls with catch-down growth were more likely to experience later pubarche (HR: 0.80, 95% CI: 0.74, 0.87; TR: 1.02, 95% CI: 1.02, 1.03), compared with the referent. This corresponds to approximately 4 months earlier and 3 months later pubic hair development, respectively (Table 3).

**Table 3:** Association Between Infant Growth Patterns and Timing of Pubarche: KPNC Puberty Study (2010–2020)

		**Unadjusted**		**Adjusted**^**a**^	
**Infant Growth Patterns**	**N**	**TR (95% CI)**	**HR (95% CI)**	**TR (95% CI)**	**HR (95% CI)**
Catch-up	4,484	0.96 (0.96, 0.97)	1.39 (1.30, 1.49)	0.97 (0.96, 0.97)	1.38 (1.28, 1.48)
Catch-down	3,837	1.02 (1.01, 1.03)	0.83 (0.77, 0.90)	1.02 (1.02, 1.03)	0.80 (0.74, 0.87)
Normal	6,562	1.00 (Reference)	1.00 (Reference)	1.00 (Reference)	1.00 (Reference)

*CI* confidence interval, *HR* hazard ratio, *KPNC* Kaiser Permanente Northern California, *TR* time ratio

^a^Adjusted for maternal age, education, prior livebirths, GWG, and girl’s birthweight, gestational age, breastfeeding duration, and race

#### Infant growth patterns and menarche

Girls with catch-up growth had 1.5 times the odds of experiencing earlier menarche (relative risk [RR]: 1.52, 95% CI: 1.36, 1.69), while girls with catch-down growth were less likely to have earlier menarche compared to the referent (RR: 0.82, 95% CI: 0.71, 0.94) (Table 4).

**Table 4:** Association Between Infant Growth Patterns and Risk of Earlier (age < 12) Menarche: KPNC Puberty Study (2010–2020)

		**Unadjusted**	**Adjusted**^**a**^
**Infant Growth Patterns**	**N**	**RR (95% CI)**	**RR (95% CI)**
Catch-up	1,873	1.56 (1.41, 1.73)	1.52 (1.36, 1.69)
Catch-down	1,655	0.83 (0.73, 0.95)	0.82 (0.71, 0.94)
Normal	2,861	1.00 (Reference)	1.00 (Reference)

*CI* confidence interval, *KPNC* Kaiser Permanente Northern California, *RR* relative risk

^a^Adjusted for maternal age, education, prior livebirths, GWG, and girl’s birthweight, gestational age, breastfeeding duration, and race

### Secondary analyses﻿

#### Effects of weight change at different periods in early life

Change in weight-for-age z-scores between birth and 2 months was associated with risk of earlier thelarche (HR: 1.12, 95% CI: 1.04, 1.21), earlier pubarche (HR: 1.17, 95% CI: 1.08, 1.28), and earlier menarche (RR: 1.08, 95% CI: 0.96, 1.23). Change in z-scores between 2 and 9 months were also associated in the thelarche (HR: 1.11, 95% CI: 1.03, 1.20), pubarche (HR: 1.11, 95% CI: 1.03, 1.21), and menarche (RR: 1.22, 95% CI: 1.09, 1.36) models. Association between growth patterns between 9–19 months and pubertal onset were weakest (Table 5).

**Table 5:** Association Between Infant Growth Patterns and Puberty at Different Early Age Periods: KPNC Puberty Study (2010–2020)

	**Thelarche**^**a**^		**Pubarche**^**a**^		**Menarche**^**a**^	
**Change in weight z-scores**	**N**	**HR (95% CI)**	**N**	**HR (95% CI)**	**N**	**RR (95% CI)**
Birth to 2 months	2,784	1.12 (1.04, 1.21)	2,772	1.17 (1.08, 1.28)	1,136	1.08 (0.96, 1.23)
2 to 9 months	2,784	1.11 (1.03, 1.20)	2,772	1.11 (1.03, 1.21)	1,136	1.22 (1.09, 1.36)
9 to 19 months	2,784	1.03 (0.95, 1.12)	2,772	1.07 (0.97, 1.17)	1,136	1.08 (0.95, 1.24)

*CI* confidence interval, *HR* hazard ratio, *KPNC* Kaiser Permanente Northern California, *RR* relative risk

^a^Adjusted for maternal age, education, prior livebirths, GWG, and girl’s birthweight, gestational age, breastfeeding duration, and race

#### Effect modification by race/ethnicity

There was some evidence of interaction between infant growth and race/ethnicity in pubarche models (*p* = 0.06). Among Black girls, those with catch-up growth had almost twice the risk of experiencing earlier pubarche compared to those with normal growth (HR: 1.69, 95% CI: 1.24, 2.32). Similarly, risk of earlier pubarche was also greater in girls experiencing catch-up growth among API (HR: 1.42, 95% CI: 1.20, 1.67), White (HR: 1.38, 95% CI: 1.22, 1.56), and Hispanic (HR: 1.23, 95% CI: 1.06, 1.42) subsets but to a lesser extent based on the point estimates. These results correspond with approximately 6, 4, 4, and 2 months earlier pubarche compared to the referent. Girls with catch-down growth were more likely to experience delayed pubarche, however associations were not significant among Asian/Pacific Islander girls (Table 6).

**Table 6:** Association Between Infant Growth Patterns and Timing of Pubarche, Stratified by Race/Ethnicity: KPNC Puberty Study (2010–2020)

	**White**			**Black**			**Hispanic**			**Asian/Pacific Islander**			**Other/Unknown**		
**Infant Growth Patterns**															
	**N**	**HR**^**a**^	**95% CI**	**N**	**HR**^**a**^	**95% CI**	**N**	**HR**^**a**^	**95% CI**	**N**	**HR**^**a**^	**95% CI**	**N**	**HR**^**a**^	**95% CI**
Catch-up	1,437	1.38 (1.22, 1.56)		375	1.69 (1.24, 2.32)		1,321	1.23 (1.06, 1.42)		970	1.42 (1.20, 1.67)		381	1.76 (1.35, 2.30)	
Catch-down	1,504	0.80 (0.70, 0.92)		170	0.52 (0.35, 0.78)		853	0.78 (0.66, 0.92)		963	0.87 (0.73, 1.03)		347	0.85 (0.64, 1.13)	
Normal	2,487	1.00 (Reference)		351	1.00 (Reference)		1,583	1.00 (Reference)		1,580	1.00 (Reference)		561	1.00 (Reference)	

*CI* confidence interval, *HR* hazard ratio, *KPNC* Kaiser Permanente Northern California

^a^Adjusted for maternal age, education, prior livebirths, GWG, and girl’s birthweight, gestational age, and breastfeeding duration

#### Mediating role of childhood BMI

Mediation by BMI was observed for the associations between catch-up growth (vs. normal) and thelarche and between catch-down growth (vs. normal) and thelarche; the percentages mediated by BMI were 67.8% (95% CI: 40.5, 95.1) and 71.8% (95% CI: 34.7, 100), respectively. Childhood BMI had a weaker mediating effect in pubarche models (catch-up: 43.6%, 95% CI: 29.0, 58.3; catch-down: 45.5%, 95% CI: 22.7, 68.3) and menarche models (catch-up: 55.5%, 95% CI: 40.4, 70.7), with the exception of catch-down growth and menarche, which was fully mediated (100%, 95% CI: 22.4, 100).

## Discussion

### Principal findings

In this racially and ethnically diverse cohort of girls, we observed that catch-up growth from birth to 24 months, and especially between birth and 9 months, was associated with earlier pubertal onset, while catch-down growth was inversely associated. These associations were independent of important confounders such as maternal age, education, prior livebirths, GWG, girl’s birthweight, gestational age, breastfeeding status, and race/ethnicity. Childhood BMI partially mediated observed associations. These data suggest that there may be other mechanisms between early life growth patterns and timing of puberty independent of childhood BMI.

### Strengths of the study

A major strength of the current study is the use of the EHR system, which allowed us to build a large longitudinal birth cohort that would have otherwise taken many years and tremendous amount of resources to conduct. Additionally, all the data were clinically and objectively assessed, including infant weight and length measures, pubertal stage data assessed by pediatricians, maternal weights before and during pregnancy, and other demographic and clinical data. Availability of these data and large and diverse study population extends and strengthens the evidence base for identified risk factors of early puberty by using a large, diverse cohort of mother-daughter pairs.

### Limitations of the data

There are limitations to this study that are worth noting. First, reliance on data from the EHR system meant that detailed data were not available on potentially relevant factors. For instance, we did not have measurements of biomarkers, diet or physical activity. Second, we did not have exact date of menarche as we used well-child questionnaire data that provided binary responses only and as such had a greater chance of outcome misclassification. Third, over 50% of the cohort was right-censored, or did not have information on age at SMR 2 + .

These girls were still very young at their most recent breast (mean 8.1 years, standard deviation = 1.5) and pubic hair (mean = 8.4, standard deviation = 1.6) SMR assessments. Heavy censoring can result in lower statistical power and precision, as reflected by wider confidence intervals which were not observed in our primary analyses. Additionally, associations were unchanged when only considering girls with interval-censored outcomes (Table 7), therefore high rates of right-censoring did not impact the results of the current study. Lastly, there was moderate imprecision in estimations of mediation effects, as seen by the wide confidence intervals.

**Table 7:** Unadjusted Associations Between Infant Growth Patterns and Pubertal Onset in Interval-Censored Girls: KPNC Puberty Study (2010–2020)

	**Thelarche**		**Pubarche**	
**Infant Growth Patterns**	**N**	**HR (95% CI)**	**N**	**HR (95% CI)**
Catch-up	1,250	1.28 (1.17, 1.40)	1,062	1.35 (1.23, 1.49)
Catch-down	1,049	0.86 (0.79, 0.94)	775	0.84 (0.75, 0.93)
Normal	1,850	1.00 (Reference)	1,486	1.00 (Reference)

*CI* confidence interval, *HR* hazard ratio, *KPNC* Kaiser Permanente Northern California

### Interpretation

In the United States the average age at menarche (12 to 13 years) has remained fairly constant for several decades [49]. However, U.S. girls are now experiencing earlier thelarche and pubarche. Compared to a seminal study published by Herman-Giddens in 1997 [50], our 2013 study demonstrated that US girls are experiencing thelarche up to two years earlier [1]. Childhood obesity is a known predictor of pubertal timing in girls, however, it does not fully explain the trend toward early puberty, as children with normal BMI are also experiencing earlier pubertal onset [6].

Several previous studies have demonstrated that girls with catch-up growth from birth to about age 2 years experienced earlier pubertal timing [8, 9, 12, 51]. In a longitudinal study of 215 German children, those with catch-up growth between birth and 24 months experienced earlier puberty (measured as pubertal growth spurt, age at peak height velocity, and menarche), all independent of pre-pubertal BMI [9]. A more recent population-based cohort study in Denmark also found that an increase in weight z-score from 0 to 12 months was associated with earlier pubertal development (self-reported SMR and other hallmarks) [8]. In a racially and ethnically diverse cohort of 262 girls in New York, Terry et al. reported that catch-up growth from ages 4 months to 1 year and from ages 1 year to 7 years were associated with earlier age at menarche [12]. Similarly, in a UK-based prospective study of 2,715 girls, Ong et al., reported that catch-up growth between 0 and 2 months and also 2 and 9 months were associated with earlier age at menarche, while subsequent weight gain between 9 and 19 months was not associated with age at menarche. Our findings are consistent with and expand on these observations by using clinician-assessed longitudinal SMR data and including multiple pubertal markers in a large, ethnically diverse sample.

 Our study may also provide some knowledge regarding a potential source of racial/ethnic differences in the timing of pubertal development. Our recent study reported that median age of thelarche among Black girls is 8.8 years, compared to 9.7 years among white girls, nearly a one-year difference [1]. Menarche among Black girls used to occur later than white girls less than a century ago [52, 53]. Perinatal factors such as excessive GWG and catch-up growth appear to affect minority populations disproportionately [54–56], as we also demonstrated in our data. The differences in the prevalence of these underlying factors may at least partially explain the striking racial/ethnic differences in the average timing of pubertal maturation.

The associations between infant growth and pubertal onset may be explained by a few potential underlying mechanisms. In the current study we found that 44–100% of the associations were mediated by childhood BMI, a proxy for adiposity. Girls with greater percent body fat are more likely to have higher concentrations of leptin and insulin: two metabolic hormones that may alter sexual development by regulating the hypothalamic-pituitary–gonadal and hypothalamic–pituitary–adrenal axes [57, 58]. Estrogen produced in fat cells may also trigger an earlier pubertal onset [2]. Some studies also suggest that individuals born small for gestational age experience an enhanced and/or prolonged ‘minipuberty’ – a temporary activation of the hypothalamic-pituitary–gonadal axis in the first year of life—compared to those born appropriate for gestational age [59, 60]. Size for gestational age and infant growth patterns are highly correlated and these early life growth-related factors may influence the programming of the hypothalamic-pituitary–gonadal axis and its functionality later in life.

## Conclusions

Our findings provide important information for clinicians and parents that girls who experience catch-up growth during this susceptible period may be at higher risk of early pubertal development. High risk girls may benefit from maintaining healthy weight through healthy diet and physical activities as childhood obesity is a known and modifiable risk factor of early puberty. Further research is needed to identify other mechanisms through which early-life growth is associated with earlier puberty.

## Data Availability

The datasets generated and/or analyzed during the current study are not publicly available due to our institutional policy. Individuals who are interested in accessing the data may contact the corresponding author regarding [or to discuss or set up] a data use agreement.

## Abbreviations

API: Asian & Pacific Islander
BMI: Body Mass Index
CI: Confidence Interval
EHR: Electronic Health Record
GWG: Gestational Weight Gain
HR: Hazard Ratio
KPNC: Kaiser Permanente Northern California
RR: Relative Risk
SMR: Sexual Maturity Rating
TR: Time Ratio
